# Predictors of quality-of-life improvement following pulmonary resection due to lung cancer

**DOI:** 10.1590/S1516-31802007000100009

**Published:** 2007-01-04

**Authors:** Ivete Alonso Bredda Saad, Neury José Botega, Ivan Felizardo Contrera Toro

**Keywords:** Quality of life, Neoplasms, Medical oncology, Rehabilitation, Questionnaires, Qualidade de vida, Câncer, Oncologia, Reabilitação, Questionários

## Abstract

**CONTEXT AND OBJECTIVE:**

There is increasing involvement of health professionals in organizing protocols to determine the impact of lung surgery on functional state and activities of daily living, with the aim of improving quality of life (QoL). The objective of this study was to investigate predictors of QoL improvement among patients undergoing parenchyma resection due to lung cancer.

**DESIGN AND SETTING:**

Prospective study, at teaching hospital of Universidade Estadual de Campinas (Unicamp).

**METHODS:**

36 patients with lung cancer diag nosis were assessed before surgery and on the 30^th^,90^th^ and 180^th^ days after surgery. The Short-Form Health Survey (SF-36) was used as the dependent variable. The independent variables were the Hospital Anxiety and Depression (HAD) scale, a six-minute walking test (6-MWT), a visual analogue scale for pain, forced vital capacity (FVC), type of surgery and use of radiotherapy and chemotherapy. Generalized estimation equations (GEE) were utilized.

**RESULTS:**

The median age for these 20 men and 16 women was 55.5 ± 13.4 years. Both FVC and 6-MWT were predictors of improvement in the physical dimensions of QoL (p = 0.011 and 0.0003, respectively), as was smaller extent of surgical resection (p = 0.04). The social component of QoL had improved by the third postoperative month (p = 0.0005).

**CONCLUSION:**

The predictors that affected QoL positively were better FVC and 6-MWT results and less extensive lung resection. Three months after the surgery, an improvement in social life was already seen.

## RESUMO

**CONTEXTO E OBJETIVO:**

Há um crescente envolvimento dos profissionais da saúde na organização de protocolos que determinem o impacto da cirurgia torácica sobre o estado funcional e as atividades de vida diária, auxiliando na melhora da qualidade de vida (QV). O objetivo deste estudo foi investigar preditores de melhora na qualidade de vida em pacientes submetidos à ressecção de parênquima pulmonar por meio do questionário Short-Form Health Survey (SF-36).

**TIPO DE ESTUDO E LOCAL:**

Estudo prospectivo realizado no Hospital das Clínicas da Universidade Estadual de Campinas, Brasil.

**MÉTODO:**

36 pacientes com câncer de pulmão foram avaliados no pré-operatório e no 30^o^, 90^o^ e 180^o^ dias do período pós-operatório. O SF-36 foi usado como variável dependente e as variações independentes foram: a escala hospitalar de ansiedade e depressão (HAD), teste de caminhada de seis minutos (TC6), escala analógica visual da dor, capacidade vital forçada (CVF), tipo de cirurgia, radioterapia e ou quimioterapia. Como modelo estatístico utilizaram-se as equações de estimação generalizadas (GEE).

**RESULTADOS:**

Foram estudados 20 pacientes do sexo masculino e 16 do feminino, idade mediana de 55.5 anos. A CVF e o TC6 foram preditores de melhora da QV nas dimensões físicas do SF-36 (p = 0,011 e p = 0,0003), bem como o tipo de ressecção cirúrgica (p = 0,04). Após três meses da cirurgia, observou-se melhora do componente social da QV (p = 0,0005).

**CONCLUSÃO:**

Os preditores de melhora da QV foram: pacientes com melhores resultados nas medidas da CVF e TC6 e aqueles com menor ressecção cirúrgica. Já no terceiro mês após a cirurgia observou-se melhora no componente social da qualidade de vida.

## INTRODUCTION

In 2001, lung cancer was responsible for more than one million deaths around the world, and was increasing at an overall rate of 5% per year, mainly at the expense of increases among women. In Brazil, few patients have the opportunity of undergoing surgery for this disease. Information on how such surgery might affect our patients’ lives is lacking in the scientific literature.^1-5^

Quality of life (QoL) is a subjective concept and assumes a customized ideal level of values, skills, satisfaction and wellbeing. Standardized questionnaires provide an objective evaluation of several characteristics involved in QoL. These questionnaires seek clear and objective answers to several questions from the multiprofessional team, relating to the effects of clinical or surgical therapy on survival and the quality of this survival.^5-8^ One of the most-used QoL questionnaires is the Medical Outcomes Study 36-item Short-Form Health Survey (SF-36). It has been used in clinical research and epidemio-logical studies because it is easy to apply and is easily understood by patients.^9^

## OBJECTIVE

The aim of this study was to investigate predictors of QoL improvement among patients who underwent pulmonary parenchyma resection due to lung cancer and who participated in a pulmonary physiotherapy program.

## METHODS

The present prospective study was performed at the Hospital das Clínicas of Universidade Estadual de Campinas, in Brazil. Between October 2001 and December 2003, 82 patients underwent pulmonary parenchyma resection due to lung cancer. Among them, nine died after surgery, 30 lived in distant places, which prevented their participation in a physiotherapy program including periodic assessments, and seven underwent palliative pulmonary resection and were not included in this study. The final study population consisted of 36 consecutive adult patients (20 males and 16 females) with a histological diagnosis of lung cancer, who underwent pulmonary parenchyma resection. All participants in the study gave their written informed consent.

The assessment protocol comprised the following instruments: SF-36, the Hospital Anxiety and Depression (HAD) scale,^10^ a spirometry test (forced vital capacity, FVC) and a six-minute walking test (6-MWT).^11^ Assessments were carried out before surgery and on the 30^th^, 90^th^ and 180^th^ days after the operation. The patients received a manual of respiratory physiotherapy for thoracic surgery and were taught to practice the exercises, beginning before the operation and continuing afterwards.

The SF-36 questionnaire generates scores on eight subscales: in the first three scales (physical functioning, physical role and bodily pain), the physical aspects of health predominate and, in the last three (social functioning, emotional role and mental health), mental aspects predominate. The scoring for each scale can vary from 0 to 100, and the greater the value is, the better the individual's performance is. A validated Portuguese version of this instrument was used.^12^

HAD is a self-reporting scale that evaluates anxiety and depression in situations where there is physical morbidity.^13^ It comprises 14 multiple-choice questions. There are two subscales of anxiety and depression, each with seven items. The overall score for each subscale ranges from 0 to 21. Scores greater than 7 were taken to define cases of anxiety/depression, in accordance with a validation study carried out in Brazil.^14^

In the spirometry test, it was considered that the FVC was abnormal when it was less than 80% of the expected value. The criteria adopted by the American Thoracic Society (1991)^15^ were used in the 6-MWT^11^ and the visual analogue scale (VAS) for pain.

Generalized estimation equations (GEE) were used for statistical analysis. QoL measured by the SF-36 questionnaire was the dependent variable. Sex, age, education, type of surgery, use of chemotherapy and/or radiotherapy, 6-MWT, FVC, HAD and level of pain were considered to be independent variables. The significance level adopted for statistical tests was 5%.^16^

## RESULTS

Table 1 shows some characteristics of the patients whose median age was 55.5 years (standard deviation, SD = 13.4).

**Table 1.** Demographic and surgical characteristics of lung-operated patients

Characteristics	Number of patients
**Sex**	
Male	20
Female	16
**Age (years)**	
≤ 39	4
≥ 40	32
**Type of surgery**	
Segmentectomy	6
Lobectomy	17
Bilobectomy	3
Pneumonectomy	10
**Type of tumor**	
Adenocarcinoma	10
Epidermoid	10
Carcinoid	6
Sarcoma	2
Metastatic carcinoma	8

Taking the preoperative period as a comparison parameter, the GEE analysis showed a decrease in QoL with regard to Physical Functioning and Physical Role on the 30^th^ day after the surgery, as would be expected, but no differences on the 90^th^ and 180^th^ day follow-up evaluations (Table 2). There were improvements on the 90^th^ and 180^th^ postoperative days in relation to the Social functioning component of SF-36.

**Table 2.** Descriptive analysis of mean values for preoperative and postoperative assessments, for each Short-Form Health Survey-36 (SF-36) component (n = 36) in lung-operated patients

SF-36 scales	Physical functioning	Physical role	Bodily pain	General health	Vitality	Social functioning	Emotional role	Mental health
**Time of Assessment**								
Preoperative	87.22	55.56	77.14	66.50	67.50	68.13	55.47	58.22
Postoperative 30 days	60.42*	19.44†	58.17	71.67	64.72	65.97	46.28	61.44
Postoperative 90 days	75.57	40.71	70.03	69.34	63.60	78.66‡	63.80	63.70
Postoperative 180 days	81.39	50.00	74.08	67.94	66.11	81.18§	64.76	61.67

*Generalized estimation equations (GEE analysis); Statistically significant results taking the preoperative time as comparison parameter:*

* *p = 0.006;*

† *p = 0.02;*

‡ *p = 0.0005; §p = 0.01.*

Using the GEE, it could be seen that patients who had undergone minor pulmonary parenchyma resection presented better QoL on the Physical Role scale of SF-36 (p = 0.04). Nineteen patients who had undergone chemotherapy and/or radiotherapy presented a decrease in QoL in the domains of Physical Functioning (p = 0.004), Physical Role (p = 0.024) and Vitality (p = 0.025).

Table 3 shows the mean values for 6-MWT and FVC before the operation and on the 180^th^ day after surgery. FVC was a predictor of improvement in the Physical Functioning component of SF-36 (p = 0.011). The 6-MWT was a predictor of improvement in QoL in the domains of Physical Functioning (p = 0.003), Physical Role (p = 0.0002) and General Health (p = 0.03) (Table 4). Neither the level of anxiety nor the level of depression was a predictor of improvement in QoL for these patients. Although seven patients were illiterate and only one had graduated from university there was no association between schooling and QoL after surgery.

**Table 3.** Mean values for forced vital capacity (%) and the six-minute walking test in lung-operated patients (n = 36)

	Preoperative	Postoperative (6-month follow-up)
Forced vital capacity	98.5% (SD ± 18)	81.5% (SD ± 23)
Six-minute walking test	509 meters (SD ± 99.5)	506 meters (SD ± 95)

*SD = Standard deviation; T-tests statistically not significant.*

**Table 4.** Analysis of GEE (generalized estimation equations) taking the Short Form-36 (SF-36) components as dependent variables and the forced vital capacity (FVC) and 6- minute walking test (6-MWT) as independent variables (only the statistically significant results are shown)

SF-36 Component (preoperative versus 180^th^ postoperative day) Variables	Estimate	Standard error	z	^p^
Physical functioning			
Forced vital capacity	0.01	0.002	2.541	0.011
Six-minute walking test	0.001	0.000	2.879	0.003
Physical role			
Six-minute walking test	0.001	0.000	3.708	0.000
General Health			
Six-minute walking test	0.001	0.000	2.108	0.031

## DISCUSSION

Overall, this study confirms the improvement in physical condition that occurs around 3-6 months after surgery.^6,17,18^ Our findings show that the predictors that positively affected the physical components of QoL were better performance in FVC and 6-MWT and less extensive lung resections. Undergoing chemotherapy and radiotherapy decreased the QoL. An improvement in the social component of SF-36 could already be seen three months after surgery.

Some methodological points need to be made. Our follow-up evaluation took the values from the preoperative period as the comparison parameter, when patients may have been in considerable physical and emotional distress. Although our study had the merit of following up 36 patients after the surgery, the improvement observed in some QoL components cannot be dissociated from the surgical recovery itself. Another point relates to the number of patients who could not be included in the study, and also the nature and severity of the diseases, which may be more serious at a tertiary-level teaching hospital than at other health facilities. In spite of these limitations, this descriptive study was carried out prospectively and may open up new hypotheses for future research.

In previous studies on QoL and lung cancer, the patients’ mean ages were higher than in our study: 67 years for Montazeri et al.^19^ (2003), 62 years for Handy et al.^6^ (2002) and 61 years for Lheureux et al. (2004).^20^ Comparing the mean ages found by these authors with what was found in the present study group, it might be asked whether our population was getting sick earlier.

Patients who undergo pneumonectomy present greater risk of complications than do those who undergo lobectomy. Moreover, mortality is a complication related to greater extent of pulmonary parenchyma resection.^3^ This negatively affects the QoL. Contrary to our results, Handy et al.^6^ (2002) did not consider that the type of surgery was a predictor of worse QoL.

Therapeutic approaches involving radio-therapy and chemotherapy have contributed towards greater or lesser survival, although the collateral effects relating to these therapies can produce several symptoms, such as general fatigue. These symptoms are associated with several physical and emotional dimensions that affect QoL.^8,19,21^ In a study by Kastersky and Paesmans^8^ (2001), QoL was not adversely affected in patients who underwent chemothe rapy because of lung cancer. Furthermore, these authors concluded that the benefits obtained through chemotherapy seemed to be followed by several positive aspects of QoL. Comparing functional state and QoL by means of SF-36, Handy et al.^6^ (2002) observed that adjuvant treatment with chemotherapy did not adversely affect QoL among patients who were followed up for six months after lung resection.

Pulmonary function has been incorporated into surgical assessments in order to predict morbidity and mortality risks following pulmonary parenchyma resection. Some authors have identified alterations in FEV_1_ (forced expiratory volume in one second) and/or FVC as possible increases in complications.^20,22^ In the present study, FVC was a predictor of significant improvement in QoL in the domain of physical functioning, although other researchers have not found an association between QoL and pulmonary function, by using the SF-36 questionnaire.^6^

Sarna et al.^17^ (2004) followed up lung cancer patients for five years and evaluated QoL by means of SF-36 and spirometric assessment. They observed that patients presented more complaints relating to respiratory symptoms such as dyspnea, productive cough and wheezing than to spirometric alterations: 36% of the patients presented moderate to severe restrictions, 24% mild restrictions and 23% had no spirometric alterations.

In the present investigation, analysis of the 6-MWT showed very similar values between the preoperative and postoperative periods (Table 2). Handy et al.^6^ found a mean value of 414 ± 107 meters. Nevertheless, for those authors, contrary to our findings, this variable was not a predictor for postoperative QoL improvement.

Our findings suggest factors that are preoperative predictors of improvement or worsening of QoL. These should be made clear to patients before the operation, in order to decrease their concerns about the surgery. The possibility that the postoperative evolution over the first month may be accompanied by physical and/or respiratory impairment should also be given consideration. Patients should participate in rehabilitation programs that can prevent or minimize physical inactivity, fatigue and lack of energy. Through this, the deleterious effects of the disease can be ameliorated and the therapeutic procedures adopted for lung cancer patients can be improved.^15,23^

Physiotherapy for patients who have undergone lung surgery consists of pulmonary ventilation exercises with emphasis on diaphragm muscle reeducation and mucociliary clearance.^24,25^ It has been proven that such exercises increase patients’ independence and self-esteem and reduce their comorbidities due to inactivity.^23,24^

## CONCLUSION

Application of the SF-36 questionnaire in the present study showed that the predictors that affected QoL positively were better FVC and 6-MWT results and less extensive lung resection. Three months after the surgery, the patients already presented quite good QoL.
